# Recent Progress of Benzodifuran‐Based Polymer Donors for High‐Performance Organic Photovoltaics

**DOI:** 10.1002/smsc.202200006

**Published:** 2022-03-23

**Authors:** Xiaoming Li, Yan Li, Yong Zhang, Yanming Sun

**Affiliations:** ^1^ School of Chemistry Beihang University Beijing 100191 P. R. China; ^2^ School of Materials Science and Engineering Harbin Institute of Technology Harbin 150001 P. R. China

**Keywords:** benzo[1,2-b:4,5-b′]difuran, fullerenes, nonfullerene acceptors, polymer solar cells, power conversion efficiencies

## Abstract

At present, a variety of benzo[1,2‐b:4,5‐b′]dithiophene (BDT)‐based polymer donors are designed and synthesized, which have made great progress toward achieving high power conversion efficiencies (PCEs) of polymer solar cells (PSCs). Despite many advantages of benzo[1,2‐b:4,5‐b′]difuran (BDF) compared with BDT unit, the overall performance of BDF polymer‐based PSCs lags far behind that of the counterpart BDT‐based polymers. Recently, great advances have been achieved in BDF polymer‐based PSCs with the highest PCEs over 16%, suggesting that BDF‐based polymers have the significant potential to catch up or even surpass the performance of BDT‐based polymers. In this review, the recent advances of BDF polymers in PSCs are first summarized and then the design strategies and the chemical structure–performance relationships of BDF polymers are discussed. Finally, perspectives on the future development of BDF polymers for high‐efficiency PSCs are provided.

## Graphical abstract

The benzo[1,2‐b:4,5‐b′]difuran (BDF) polymer‐based organic solar cells (OSCs) have made great progress and achieved high power conversion efficiencies (PCEs) over 16%. To further explore high‐performance BDF‐based polymer donors, the recent advances with the innovation of BDF copolymers in OSCs are summarized. Meanwhile, the design strategies between the chemical structure and photovoltaic performance of BDF‐based polymers are discussed.

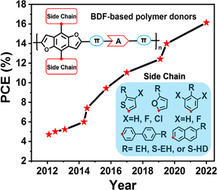

## 1 Introduction

Polymer solar cells (PSCs) provide an alternative and innovative method for harvesting solar energy, which is a fully renewable source, holding great promise for fabricating lightweight and flexible devices through solution‐processing techniques.^[^ ^1^, ^2^, ^3^, ^4^, ^5^, ^6^ ^]^ In the past few years, the record power conversion efficiencies (PCEs) of single‐junction PSCs were frequently updated and increased from 10% to 18%.^[^ ^7^, ^8^, ^9^, ^10^, ^11^, ^12^, ^13^, ^14^, ^15^, ^16^, ^17^, ^18^ ^]^ The improvements of photovoltaic performance cannot be separated from explorations into novel polymer donors and nonfullerene acceptors (NFAs).^[^ ^19^, ^20^, ^21^, ^22^, ^23^, ^24^, ^25^, ^26^, ^27^, ^28^, ^29^ ^]^ Zhan and co‐workers reported ITIC NFA with an A–D–A conformation, solving the drawbacks of weak light absorption and relatively fixed energy levels of fullerene and its derivatives.^[^ ^30^, ^31^, ^32^, ^33^ ^]^ Subsequently, considering distinct advantages in terms of light absorption and variable bandgaps and energy levels, a variety of new NFA acceptors have been explored.^[^ ^34^, ^35^, ^36^, ^37^, ^38^, ^39^, ^40^ ^]^ Recently, Zou and co‐workers reported a Y6 NFA, with an A–DA'D–A conformation, which is a new category of NIR‐absorbing NFAs for high‐performance PSCs.^[^ ^15^ ^]^

**Figure 1:**
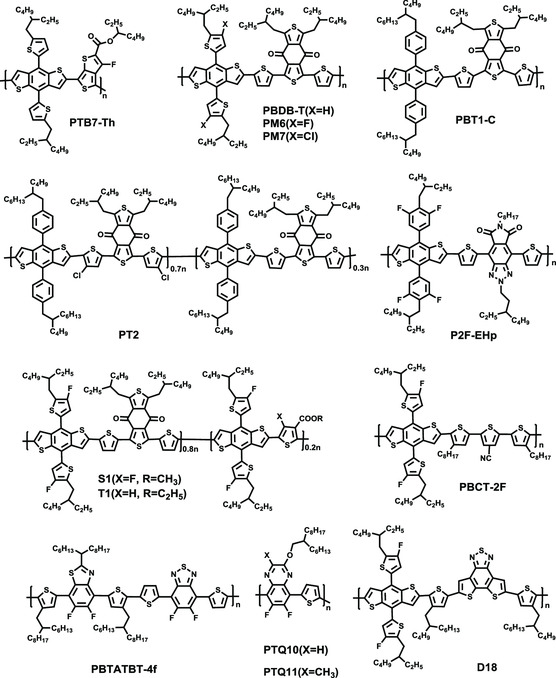
Chemical structures of high‐efficiency polymer donors for PSCs.

Typically, the organic solar cell is based on a bulk‐heterojunction (BHJ) architecture, in which a conjugated polymer serves as a donor to blend with an electron acceptor, enabling a sufficient photoinduced charge separation process.^[^ ^41^, ^42^ ^]^ Parallel to the development of fullerene and NFAs in PSCs, diverse types of polymer donors have been explored.^[^ ^43^, ^44^, ^45^, ^46^, ^47^, ^48^, ^49^, ^50^, ^51^, ^52^, ^53^, ^54^, ^55^ ^]^ To date, benzo[1,2‐b:4,5‐b′]dithiophene (BDT)‐based polymer donors have received considerable attention and shown excellent photovoltaic performance. For example, PTB7‐Th and PBDB‐T, both comprising the BDT unit, have been designed to match well with fullerenes. They also function well as the donor materials in nonfullerene systems.^[^ ^7^, ^56^, ^57^, ^58^, ^59^, ^60^ ^]^ State‐of‐the‐art polymer donors, PM6, P2F‐EHp, D18, etc., containing a BDT unit, have shown unprecedented progress in NFA‐based PSCs (NFA‐PSCs) (**Figure** **1**).^[^ ^52^, ^53^, ^61^, ^62^, ^63^, ^64^, ^65^, ^66^, ^67^, ^68^, ^69^, ^70^, ^71^, ^72^, ^73^ ^]^ Moreover, a number of high‐efficiency donor polymers also have been explored without the BDT unit, such as PTQ10, PBTATBT‐4f, etc. However, its furan analogue, benzo[1,2‐b:4,5‐b′]difuran (BDF), which possesses a smaller size than the BDT unit and thus generates weaker steric hindrance to adjacent units and tighter packing arrangement, is less developed as an efficient building block for the polymer donors. Meanwhile, the BDF unit has deeper highest occupied molecular orbit (HOMO) and the lowest unoccupied molecular orbital (LUMO) energy levels than its BDT analogue, which can effectively lower the molecular energy levels. In addition, the furan unit can be obtained easily and cheaply from numerous biorenewable products, making BDF‐based polymer donors more suitable for future practical applications.^[^ ^74^, ^75^, ^76^ ^]^ With the aforementioned discussions, BDF‐based copolymers may exhibit more eminent photovoltaic properties than those of BDT‐based copolymers. However, far less effort has been focused on the BDF polymers in comparison with BDT polymers, and only a few cases in the context of NFA‐PSCs involving BDF copolymers have been reported. Accordingly, it is worthwhile to further explore novel BDF‐based polymer donors for fabricating high‐performance NFA‐PSCs.

There have been many reviews that in detail summarize the recent development of BDT‐based polymer donors. However, the recent progress upon BDF‐based polymer donors has been rarely summarized. Considering the significant progress for BDF polymer‐based NFA‐PSCs recently, it is imperative to discuss them specifically. In this article, we briefly describe the origin of the furan‐fused unit and its applications in fullerene‐based PSCs. Then, our emphasis will be focused on the advances of BDF‐based polymers in NFA‐PSCs, including their design strategies, structure/property relationships, and photovoltaic performance. Finally, we provide design strategies to develop appropriate BDF polymers for high‐efficiency PSCs.

## 2 Early Reports of BDF‐Based Polymers in Fullerene‐Based PSCs

**Figure 2:**
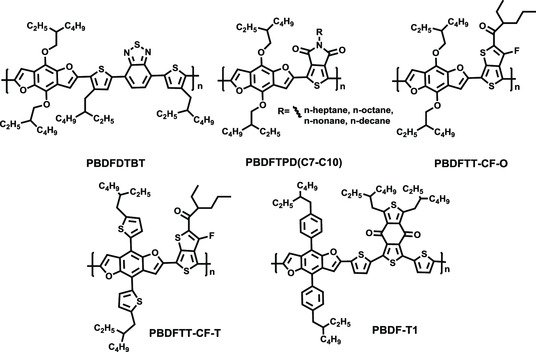
The molecular structures of BDF‐based representative polymers in fullerene‐based PSCs.

In early 2000, Kobayashi and co‐workers reported a synthetic method to afford furan‐fused quinone, benzo[1,2‐b:4,5‐b′]difuran‐4,8‐dione, but the electrical properties of the furan‐fused unit have not been explored in the photovoltaic field.^[^ ^77^ ^]^ The aromatic BDF unit possesses a good planar structure, excellent intermolecular π–π stacking, and rigid conformation, which has been applied to semiconductor materials frequently. As shown in **Figure** **2**, a low‐bandgap donor–acceptor (D–A) copolymer PBDFDTBT containing a BDF unit was first designed and synthesized until 2012, which selected 4,7‐dithienyl‐2,1,3‐benzothiadiazole (DTBT) as the electron‐withdrawing block and 4,8‐bis‐ethylhexyloxy‐benzo[1,2‐b:4,5‐b′]difuran as the electron‐sufficient unit. This polymer opens the door for the BDF unit used in photoelectric devices. As shown in **Table** **1**, PBDFDTBT:PC_71_BM devices exhibited a promising PCE of 5.01%, competing with the representative polymer donors at that time.^[^ ^78^ ^]^ Subsequently, 4,8‐bis‐ethylhexyloxy‐benzo[1,2‐b:4,5‐b′]difuran‐based copolymers with various acceptor units were designed and investigated (Figure 2). For example, Beaujuge and co‐workers synthesized a series of BDF polymers (PBDFTPD, Figure 2) with the TPD unit. As depicted in Table 1, the PBDFTPD(2EH/C8):PC_71_BM device achieved a PCE of 7.40%, with a large *V*_OC_ of 0.97 V and a high fill factor (FF) of 68%.^[^ ^79^ ^]^ Such obvious improvement in *V*_OC_ can be assigned to the low‐lying HOMO energy level. To enhance the intermolecular π–π interactions, Hou et al. used the 2‐alkylthienyl group to replace the aforementioned alkoxyl groups at four and eight positions of the BDF unit to generate the 2D‐conjugated polymer PBDFTT‐CF‐T.^[^ ^80^ ^]^ The alkoxyl side chain substituted with alkylthienyl side groups possesses a weaker electron‐donating ability, which results in a lower HOMO level in PBDFTT‐CF‐T compared with that of PBDFTT‐CF‐O. Meanwhile, the 2D‐conjugated side chain is beneficial to enhancing the intermolecular π–π stacking and improving charge transport. It can be confirmed that the hole mobility of PBDFTT‐CF‐T/PC_71_BM blend is higher than that of PBDFTT‐CF‐O/PC_71_BM blend.

**Table 1:** Optical bandgap, HOMO energy levels, and photovoltaic parameters based on BDF‐based polymers in the fullerene system

Polymers	*E*opt g [eV]	HOMO [eV]	*V*_OC_ [V]	*J*_SC_ [mA cm^−2^]	FF [%]	PCE [%]	Ref.
PBDFDTBT	1.60	−5.10	0.78	11.77	54.6	5.01	[78]
PBDFTPD(C8)	1.97	−5.41	0.97	11.20	68.0	7.40	[79]
PBDFTT‐CF‐O	1.51	−4.98	0.63	13.87	59.7	5.22	[80]
PBDFTT‐CF‐T	1.49	−5.21	0.78	13.04	61.6	6.26	[80]
PBDF‐T1	1.83	−5.43	0.92	13.28	77.4	9.43	[81]

Furthermore, we replaced thiophene side group with 2‐ethylhexylphenyl in the BDF core, and 1,3‐bis(thiophen‐2‐yl)‐5,7‐bis(2‐ethylhexyl)benzo[1,2‐c:4,5‐c′]dithiophene‐4,8‐dione (BDD) was selected as an acceptor unit to get a novel BDF‐based copolymer, PBDF‐T1 (Figure 2).^[^ ^81^ ^]^ A breakthrough of PCE as high as 9.43% was reported based on PBDF‐T1 and PC_71_BM, which represents the highest PCE value for BDF‐based conjugated polymers in the fullerene system now. Compared with the polymer PBDFTT‐CF‐T, PBDF‐T1 has a deeper HOMO level, which has more potential for realizing high *V*_OC_. This great progress demonstrated that BDF‐based polymers can be competitive with the BDT‐based polymer counterparts via rational molecular design and morphological optimization, which offers great promise for the next generation of high‐efficiency polymer donors. However, even if fullerene‐based PSCs exhibited a high FF ultimately, only limited PCE values could be achieved due to weak absorption in the visible spectral region and relatively fixed energy levels in the fullerene acceptor. Moreover, the large energy loss (*E*_loss_) remains a critical issue that limits the PCE improvement for fullerene‐based PSCs. To resolve these issues and keep pace with the rapidly developed PSCs, it is worthwhile to further explore novel BDF‐based polymer donors and obtain outstanding photovoltaic performance for NFA‐PSCs.

## 3 BDF‐Based Polymers in NFA‐PSCs

**Figure 3:**
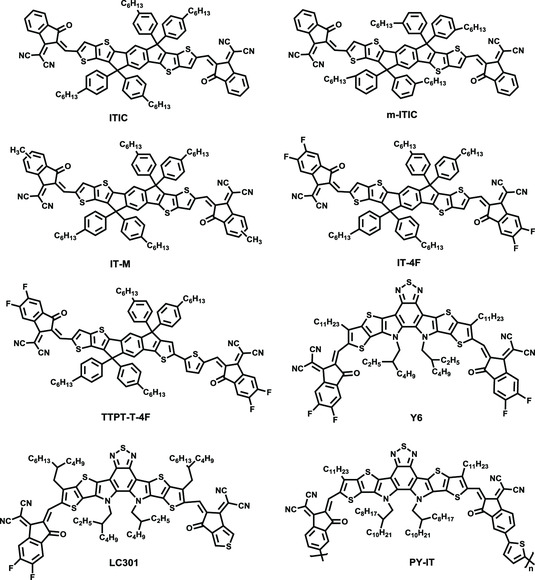
Molecule structures of representative NFAs in BDF‐based polymers.

An alternative strategy has emerged, converting low‐bandgap polymers to wide‐ or medium‐bandgap polymers to achieve good absorption complementation and matched energy levels in BDF‐based polymers for the past several years. In comparison with the number and diversity of NFAs and BDT‐based polymers, the development of BDF‐based polymers is of great urgency, particularly pairing with NFAs (**Figure** **3**) in terms of highly efficient NFA‐PSCs.

### 3.1 BDF‐Based Polymers with ITIC and Its Derivatives

**Figure 4:**
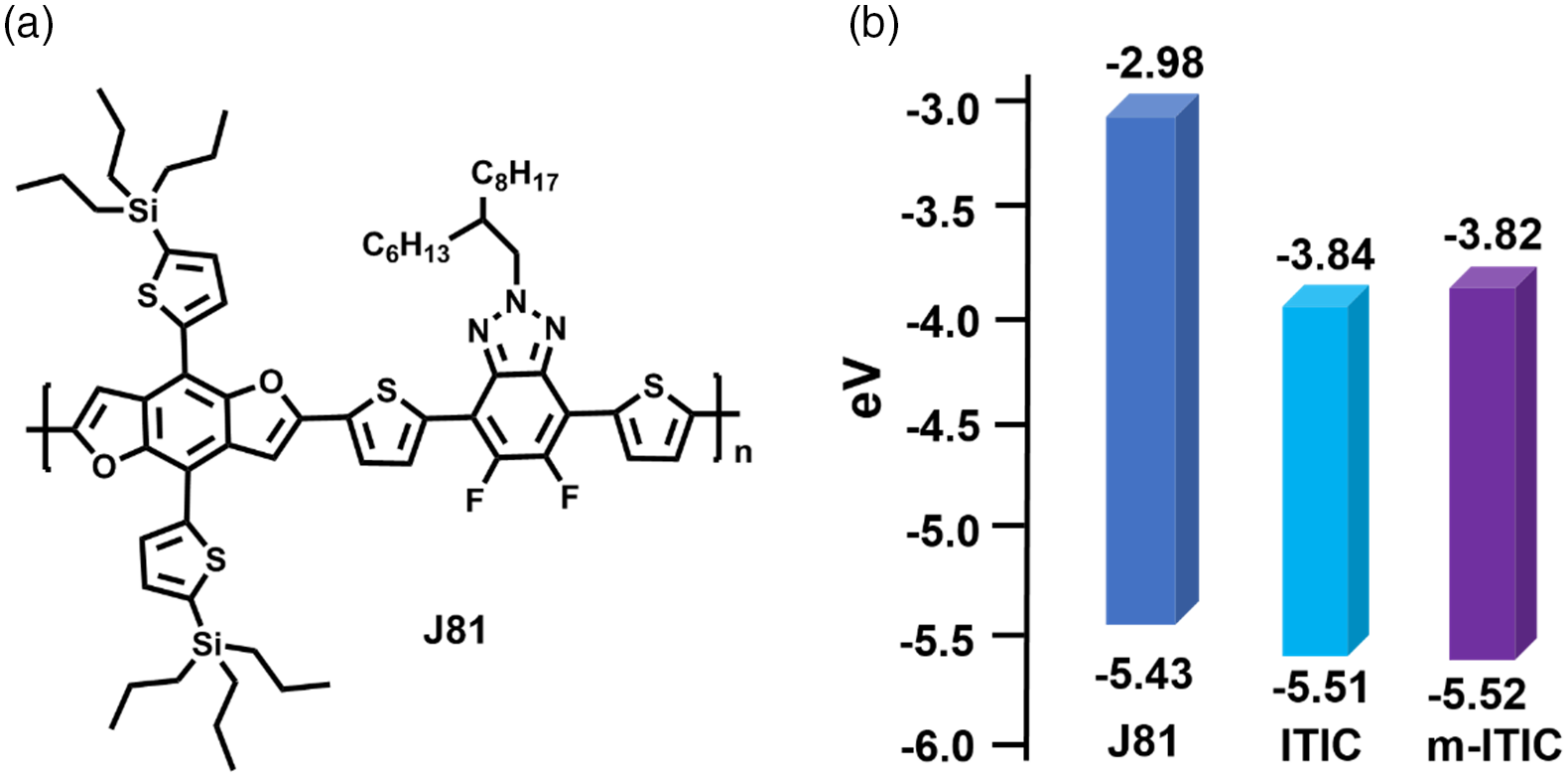
a) Molecule structure of J81. b) Energy‐level diagrams of J81, ITIC, and m‐ITIC.

Li and co‐workers synthesized a 2D‐conjugated polymer J81, introducing trialkylsilyl thiophene side chains on the BDF unit, combining difluorinated benzo[d][1,2,3]triazoles (BTz) unit, as shown in **Figure** **4** and **Table** **2**.^[^ ^82^ ^]^ J81 shows a low‐lying HOMO energy level of −5.43 eV, an absorption from 400 to 642 nm, displaying complementary absorption using ITIC or m‐ITIC as an acceptor in the visible–near infrared region. As shown in Figure 3, ITIC was developed by Zhan et al., which possesses a suitable LUMO energy level and strong absorption from 500 to 800 nm to obtain high *V*_OC_ and *J*_SC_ in the corresponding PSCs. m‐ITIC was reported with a modification on ITIC by side‐chain isomerization with meta‐alkylphenyl substitution, which shows a higher absorption coefficient and electron mobility.^[^ ^83^ ^]^ For these two acceptors, the HOMO/LUMO energy levels were similar and located at −5.51/−3.84 eV and −5.52/−3.82 eV for ITIC and m‐ITIC, respectively. It is worth noting that the HOMO level offset between J81 and ITIC/m‐ITIC was smaller (below 0.1 eV) compared with that of fullerene‐based PSCs (at least 0.3 eV), traditionally believing that devices cannot show efficient charge generation upon such small energy offset. The photoluminescence of the two blend films with J81 were efficiently quenched, generating effective electron transfer and hole transfer even through small energy offset. The small (even negligible) driving energies could reduce the voltage loss in PSCs. Therefore, there is a promising prospect for us to achieve high *J*_SC_ and large *V*_OC_ simultaneously for NFA‐PSCs and improve the PCE ultimately. As shown in Table 2, for the PSCs based on J81:ITIC and J81:m‐ITIC, the optimal PCEs reached 10.60% and 11.05%, respectively, which are much higher than the highest efficiency of 9.43% in the fullerene system. The significant improvement in PCE is mainly benefitted from the increased *V*_OC_ and *J*_SC_. The large *V*_OC_ can be attributed to small energy offset for J81 and the acceptors. As for high *J*_SC_, it is mainly because NFAs have broader absorption in the visible–near infrared region, showing complementary absorption with J81. It was a successful attempt for BDF‐based polymer donors in NFA‐PSCs, which reduced PCE differences compared with the BDT‐based polymer counterparts.

**Table 2:** Optical bandgap, HOMO levels, and photovoltaic parameters based on PBDFT‐Bz and its derivatives

Polymer	*E*opt g[eV]	HOMO [eV]	*V*_OC_ [V]	*J*_SC_ [mA cm^−2^]	FF [%]	PCE [%]	Ref.
J81	1.93	−5.43	0.95	15.27	73.1	10.60	[82]
(ITIC/m‐ITIC)			0.96	16.48	69.8	11.05	
PBDFT‐Bz (ITIC/m‐ITIC)	1.90	−5.35	0.85	15.56	70.0	9.26	[86]
			0.85	16.63	70.0	9.84	
PBDFF–Bz	1.85	−5.48	0.94	15.02	67.0	9.46	[86]
(ITIC/m‐ITIC)			0.94	16.57	66.0	10.28	
PBDFS‐Bz	1.88	−5.30	0.82	15.14	65.0	8.07	[87]
PBDFS‐fBz	1.89	−5.45	0.88	15.26	67.0	9.00	[87]
PBDFT‐FBz	1.92	−5.39	0.81	13.93	64.9	7.30	[88]
PBDFF‐FBz	1.93	−5.46	0.83	14.43	69.4	8.32	[88]
PBDFFPD	1.71	−5.52	0.84	16.27	70.1	9.58	[89]
L2(F11)	1.86	−5.50	0.86	22.17	73.6	14.00	[91]
F10(P‐FT)	1.93	−5.48	0.91	16.89	69.5	10.50	[90]
F11(P‐ClT)	1.92	−5.50	0.92	17.75	69.6	11.37	[90]
PBDFTz‐SBP	1.91	−5.41	0.89	18.56	75.2	12.42	[75]
PBDFP‐Bz	1.91	−5.50	0.97	17.83	64.2	11.10	[92]
(ITIC/IT‐M)			1.02	18.27	69.4	12.93	
P‐P	–	−5.33	0.80	16.25	56.3	8.28	[93]
F13(P–FP)	1.93	−5.54	0.96	15.72	57.7	8.86	[93]
PBDF‐BDD	–	−5.36	0.86	17.67	70.4	10.65	[94]
PBF1‐C	1.79	−5.43	0.74	17.52	71.1	9.16	[95]
PBF1‐C‐2Cl	1.97	−5.61	0.86	17.98	71.6	11.09	[95]

**Figure 5:**
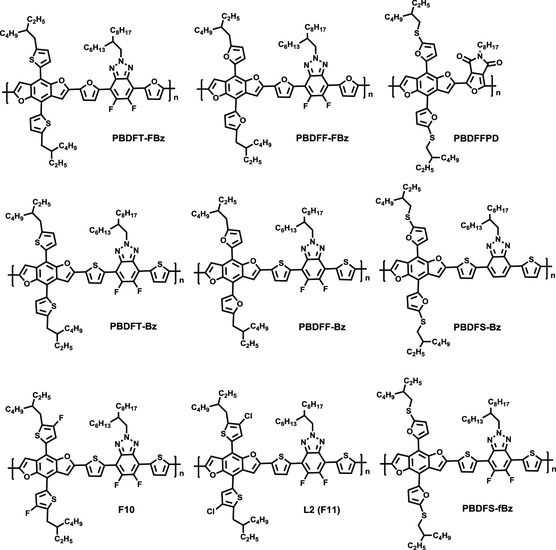
Molecular structures of PBDFT‐Bz and its derivatives.

Subsequently, a series of BDF‐based polymers with the BTz unit were reported, and their molecule structures, optical bandgap, HOMO energy levels, and photovoltaic parameters are depicted in **Figure** **5** and Table 2. Integrating the advantage of a series of wide band‐gap copolymers based on BDT and BTz, such as PBDTT–Bz (J52), J60, J61, etc., Zhang and co‐workers reported two wide‐bandgap‐conjugated polymers, PBDFT–Bz and PBDFF–Bz, respectively.^[^ ^84^, ^85^, ^86^ ^]^ Replacing 2‐ethylhexylthienyl side chains with 2‐ethylhexylfuryl side chains, PBDFF–Bz possesses a lower HOMO energy level than that of PBDFT–Bz. The corresponding PSC exhibited a *V*_OC_ of 0.94 V, which is 0.09 V higher than that of the PBDFT–Bz‐based device. Following the success of furan‐based BDF polymers, the alkythiofuran‐based BDF polymer consisting of fluorinated and nonfluorinated BTz, PBDFS‐Bz and PBDFS‐fBz, was designed, respectively.^[^ ^87^ ^]^ By introducing two fluorine atoms into the polymer PBDFS‐Bz, the HOMO energy level and the molecular spacing could be regulated. As displayed in Table 2, the *V*_OC_ of the fluorinated polymer PBDFS‐fBz‐based PSC is 0.06 V higher than that of the nonfluorinated analogue polymer PBDFS‐Bz, and finally an enhanced PCE of 9.0% was achieved. Main‐chain engineering was also adopted to alter thiophene spacing units in PBDFT‐FBz to furan spacing units in the main chain of PBDFF‐FBz, and the smaller size of furan units in PBDFF‐FBz can generate the enhanced polymer chain interactions than that of PBDFT‐FBz. The result of X‐ray diffraction (XRD) indicated that the lamellar *d*‐spacing of PBDFF‐FBz is smaller than that of PBDFT‐FBz in the (100) direction.^[^ ^88^ ^]^ Benefiting from the lower HOMO energy level and stronger chain interactions, the PBDFF‐FBz‐based PSC exhibited a higher PCE of 8.32% with enhanced *V*_OC_, *J*_SC_, and FF simultaneously. Motivated by the success of PBDFF‐FBz and PBDFS‐Bz, a medium‐bandgap copolymer PBDFFPD was designed and synthesized, which was based on the all‐furan skeleton BDF and furo[3,4‐c]pyrrole‐4,6‐dione (FPD).^[^ ^89^ ^]^ The FPD unit was synthesized via only three steps with low cost, which is desirable for the development of the low‐cost and efficient furan‐based polymer donors. The structure of PBDFFPD enables a rigid and large conjugated plane due to the multiple noncovalent interactions including C—O···S, C—O···H, C═O···H, C═O···π; thus, the polymer possesses a nearly planar conjugated skeleton with negligible torsion and strong intermolecular and intramolecular interaction. In addition, the introduction of a strong electron‐withdrawing FPD unit resulted in a low‐lying HOMO level of −5.52 eV for FPD‐based polymers, which is beneficial for obtaining a high *V*_OC_ in the corresponding PSC. In comparison with PBDFS‐Bz, the PBDFFPD‐based device achieved a PCE of 9.58%, with a prominent FF of 70.1%, an enhanced *J*_SC_ of 16.27 mA cm^−2^, and a *V*_OC_ of 0.84 V. Thus, these results exhibited that modifying backbone conformations of BDF‐based polymers could have a positive influence on improving the photovoltaic performance.

**Figure 6:**
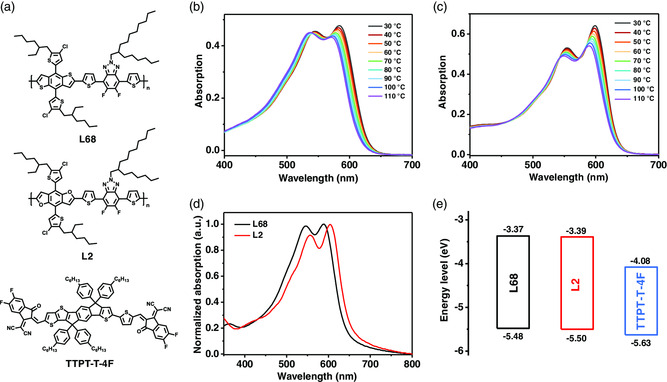
a) Chemical structures of L68, L2 and TTPT‐T‐4F. Temperature‐dependent absorption spectra in diluted chlorobenzene for b) L68 and c) L2. d) Absorption spectra of the two copolymers in the film; e) energy levels of L68, L2, and TTPT‐T‐4F. Reproduced with permission.^[^ ^91^ ^]^ Copyright 2019, Wiley‐VCH.

**Figure 7:**
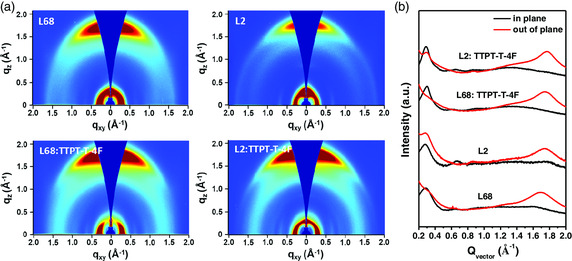
a) 2D GIWAXS patterns and b) the corresponding linecut scattering profiles of IP (black) and OOP (red) for the neat L68 and L2 films and their blended films. Reproduced with permission.^[^ ^91^ ^]^ Copyright 2019, Wiley‐VCH.

Halogenation on either donor or acceptor units of a polymer has been approved as an efficient molecular modification strategy to enhance the photovoltaic performance. To this end, we introduced the fluorine (F) and chlorine (Cl) atoms on the thienyl side chains of the BDF unit to synthesize two BDF polymers, F10 and F11 (L2). Due to the strong electronegative properties of F and Cl atoms, F10 and F11 showed lower HOMO energy levels than the nonhalogenated PBDFT‐Bz. The PSCs of F10:m‐ITIC and F11:m‐ITIC without solvent additive provided PCEs of 10.50% and 11.37%, respectively.^[^ ^90^ ^]^ The *V*_OC_ of F11:m‐ITIC device was also increased to 0.921 V (Table 2). In addition, the empty 3*d* orbitals of Cl atom could make π‐electron delocalization effective, and their interactions with F and/or hydrogen (H) will also improve the molecular order. In addition to Zhang's group, the same structure F11 was reported by Sun et al., who evaluated the performance of the BDF‐based device using TTPT‐T‐4F as an acceptor. Compared with the F11:m‐ITIC‐based device, the improvement of PCE based on L2:TTPT‐T‐4F is mainly due to the excellent *J*_SC_ and FF. As shown in **Figure** **6**, redshifted absorption and a more pronounced shoulder peak were observed for L2 (F11), suggesting that L2 had a tighter interchain packing, compared with the BDT‐based analogue polymer L68.^[^ ^91^ ^]^ Furthermore, the temperature‐dependent absorption spectra indicated that copolymer L2 showed strong aggregation behavior in the solution. L2 had slightly lower HOMO/LUMO energy levels compared with that of the counterpart L68, which were −5.50/−3.39 and −5.48/−3.37 eV, respectively. It was probably due to the higher electronegativity of oxygen than that of the sulfur atom in the BDF core. The higher hole mobility (*μ*_h_) (L2 vs L68) demonstrated that L2 possesses the more planar molecular backbone, which could be demonstrated by the grazing incident wide‐angle X‐ray scattering (GIWAXS) of neat films in **Figure** **7**. As shown in Table 2, the PSC based on L2:TTPT‐T‐4 F showed a superior PCE of 14.0%, with a *V*_OC_ of 0.86 V, a high *J*_SC_ of 22.17 mA cm^−2^, and an FF of 73.6%. The optimal device of L2 is higher than that of the L68‐based device (12.72%). When L2 and L68 blended with the acceptor TTPT‐T‐4F, the blend films exhibited preferable face‐on orientation, along with strong (010) diffraction of π–π stacking at 1.74 and 1.77 Å^−1^ in the out‐of‐plane (OOP) direction and (100) lamellar scattering in the in‐plane (IP) direction, respectively. The tighter π–π stacking (*d*_spacing_ = 3.55 Å) in the L2‐based blend would improve charge carrier transport and collection, which is consistent with better photovoltaic results.

**Figure 8:**
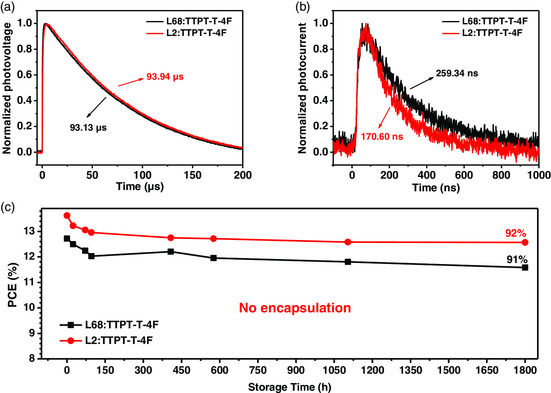
a) TPV and b) TPC curves of the L68‐ and L2‐based devices. c) Ambient stability results of the L68‐ and L2‐based devices. Reproduced with permission.^[^ ^91^ ^]^ Copyright 2019, Wiley‐VCH.

Importantly, we also investigated the nonradiative recombination loss (Δ*E*_3_ = *q* $\Delta V_{\text{OC}}^{\text{non}-\text{rad}}$) to understand the origin of the higher *V*_OC_ in L2‐based devices. Δ*E*_3_ of the device based on L2:TTPT‐T‐4F (0.334 eV) was smaller than that of the L68:TTPT‐T‐4F device (0.343 eV), suggesting that the nonradiative recombination loss could be slightly suppressed in the BDF‐based PSCs. Meanwhile, there is still a big possibility to reduce Δ*E*_3_ to improve the *V*_OC_. To corroborate the promotion of *J*_SC_ and FF and quantify the charge transport and recombination dynamics, transient photovoltage (TPV) and transient photocurrent (TPC) were measured. As shown in **Figure** **8**a, TPV measurement exhibited that the carrier lifetime (*τ*) was 93.94 and 93.13 μs for L2:TTPT‐T‐4F and L68:TTPT‐T‐4F under the open‐circuit voltage, respectively, suggesting that these polymers both exhibited similar charge recombination behaviors. Meanwhile, the faster charge sweep‐out time of 170.60 ns was obtained for the L2‐based devices from the TPC curves (Figure 8b) than that of 259.34 ns for the L68‐based devices, which indicated a more efficient charge transport and extraction. In addition, the storage tests were carried out to investigate the potential of commercial applications of BDF‐based PSCs in ambient atmosphere. As shown in Figure 8c, after storage for 1800 h in air with a relative humidity of about 42%, the L2:TTPT‐T‐4 F‐based device exhibited excellent stability and maintained 92% of its initial PCE value. Therefore, BDF‐based copolymers had the potential to show excellent photovoltaic performance when these polymers were combined with appropriate NFAs. These results also concluded that the halogen atomic substitution approach is a promising molecular design strategy to obtain state‐of‐the‐art BDF‐based polymer donors.

**Figure 9:**
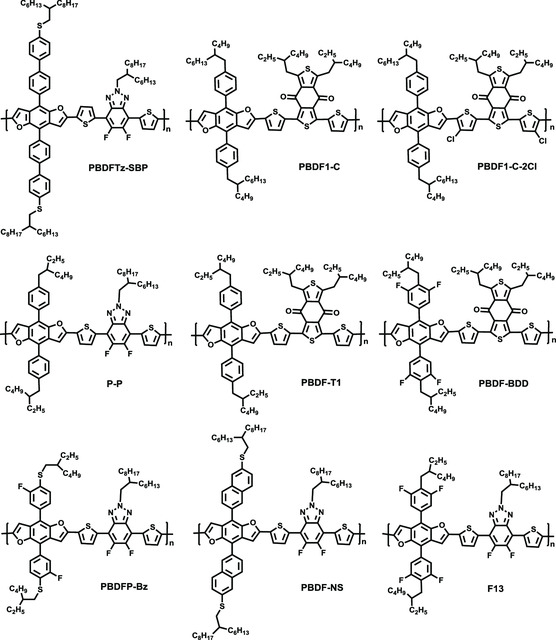
Molecular structures of PBDF‐T1 and its derivatives.

Considering the advantages of para‐alkyl‐phenyl‐substituted BDF‐based polymer PBDF‐T1 (**Figure** **9**), 2D extended biphenyl side chains on BDF as the donor unit were synthesized, which could extend the π‐conjugation length to improve the intermolecular π–π interaction further.^[^ ^75^ ^]^ The normalized absorption spectrum of PBDFTz‐SBP in *o*‐DCB solution exhibits a small vibronic shoulder peak in the longer‐wavelength region, while the absorbance shoulder gradually disappeared with the increasing solution temperature. The results indicate that the extended π–π conjugation length in the side chain may cause strong aggregation behaviors in the BDF‐based polymers, while its temperature‐dependent aggregation (TDA) property enables the well‐controlled and near‐optimal morphology. Initially, the polymer combined with ITIC only achieved an inferior PCE of 7.95% under a low‐temperature condition. It may be ascribed to the strong aggregation properties of the BDF‐based polymer, which results in large phase segregation, poor exciton dissociation, and charge collection. When preheating both the active‐layer solution and the substrate to ≈100 °C, the polymer PBDFTz‐SBP‐based PSC obtained a PCE up to 12.42%. The transmission electron microscopy results verified an aggregation‐breaking process as the temperature varied, eliminating the strong intermolecular interaction and large phase separation. The structure–property relationship could be reflected on the device performance; thus, a rational molecular design could insure BDF‐based polymers containing strong TDA property and favorable morphology.

Furthermore, a copolymer PBDFP‐Bz was designed and synthesized via fluorine and sulfur atoms to modify benzene side chain (Figure 9).^[^ ^92^ ^]^ This polymer displayed a large coplanar structure and deep‐lying HOMO energy level of −5.50 eV, and the PSC based on PBDFP‐Bz:ITIC showed an optimal PCE of 11.10% with a high *V*_OC_ of 0.97 V and a small *E*_loss_ of 0.60 eV. To further improve the *V*_OC_ and reduce energy loss of PBDFP‐Bz‐based PSCs, ITIC was replaced with IT‐M, which possesses an upward LUMO level and higher extinction coefficient. The optimized PBDFP‐Bz:IT‐M PSC displayed a remarkable PCE of 12.93% with a lower *E*_loss_ of 0.57 eV, which produced a significant *V*_OC_ of 1.02 V (Table 2). To further realize a reduced total energy loss and still maintain high PCEs in PSCs, a polymer P‐FP (F13) has the same main backbone with polymer PBDFP‐Bz, while P‐FP possesses difluorinated phenyl side chains. *E*_loss_ in P‐FP:m‐ITIC device is 0.585 eV, which is lower than that of the polymer PBDFP‐Bz‐based device, while the PCE is only 8.86% with a low *J*_SC_ and FF.^[^ ^93^ ^]^ Likewise, a fluorinated polymer PBDF‐BDD was obtained using Stille polymerization between the BDD unit and the monomer (4,8‐bis(4‐(2‐ethylhexyl)‐3,5‐difiuorophenyl)benzo[1,2‐b:4,5‐b′] difuran‐2,6‐diyl)bis(trimethylstannane). However, PBDF‐BDD‐based PSCs showed a large *E*_loss_ of 0.714 eV, thus achieving a poor PCE of 10.65% with IT‐4 F. ^[^ ^94^ ^]^ Considering the advantages of Cl incorporation and successful example in polymer L2, in 2020, our group designed and synthesized two BDF copolymers, PBF1‐C and PBF1‐C‐2Cl.^[^ ^95^ ^]^ PBF1‐C is a derivative from the state‐of‐the‐art donor polymer PBDF‐T1 in the fullerene system, which was obtained by replacing the alkyl side chain ethylhexyl with butyloctyl in phenyl side chain. Furthermore, by replacing thiophene π‐bridge with 3‐chlorothiophene in PBF1‐C, a chlorinated polymer PBF1‐C‐2Cl was obtained with a low‐lying HOMO level. Compared with a halogenation strategy in the polymer's conjugated side chains, inserting a chlorinated π‐bridge could also reduce the energy level efficiently, which is conducive to yielding a high *V*_OC_ in PSCs. Initially, IT‐4 F was selected as the acceptor to match these two copolymers. However, PBF1‐C:IT‐4 F‐based devices exhibited a PCE of 9.16%, with a low *V*_OC_ of 0.74 V, and PBF1‐C‐2Cl:IT‐4 F devices showed a higher PCE of 11.09%, and such performance promotion mainly benefits from a higher *V*_OC_ of 0.86 V. However, the best‐performing device based on PBF1‐C‐2Cl only attained an ordinary *J*_SC_ of 17.98 mA cm^−2^; thus, we selected the state‐of‐the‐art NFA Y6 as the acceptor to investigate whether they could achieve more excellent photovoltaic performance. In the following sections, the photovoltaic properties of some aforementioned BDF copolymers with Y6 will be discussed in detail, respectively.

### 3.2 BDF‐Based Polymers with Y6 and Its Derivatives

**Figure 10:**
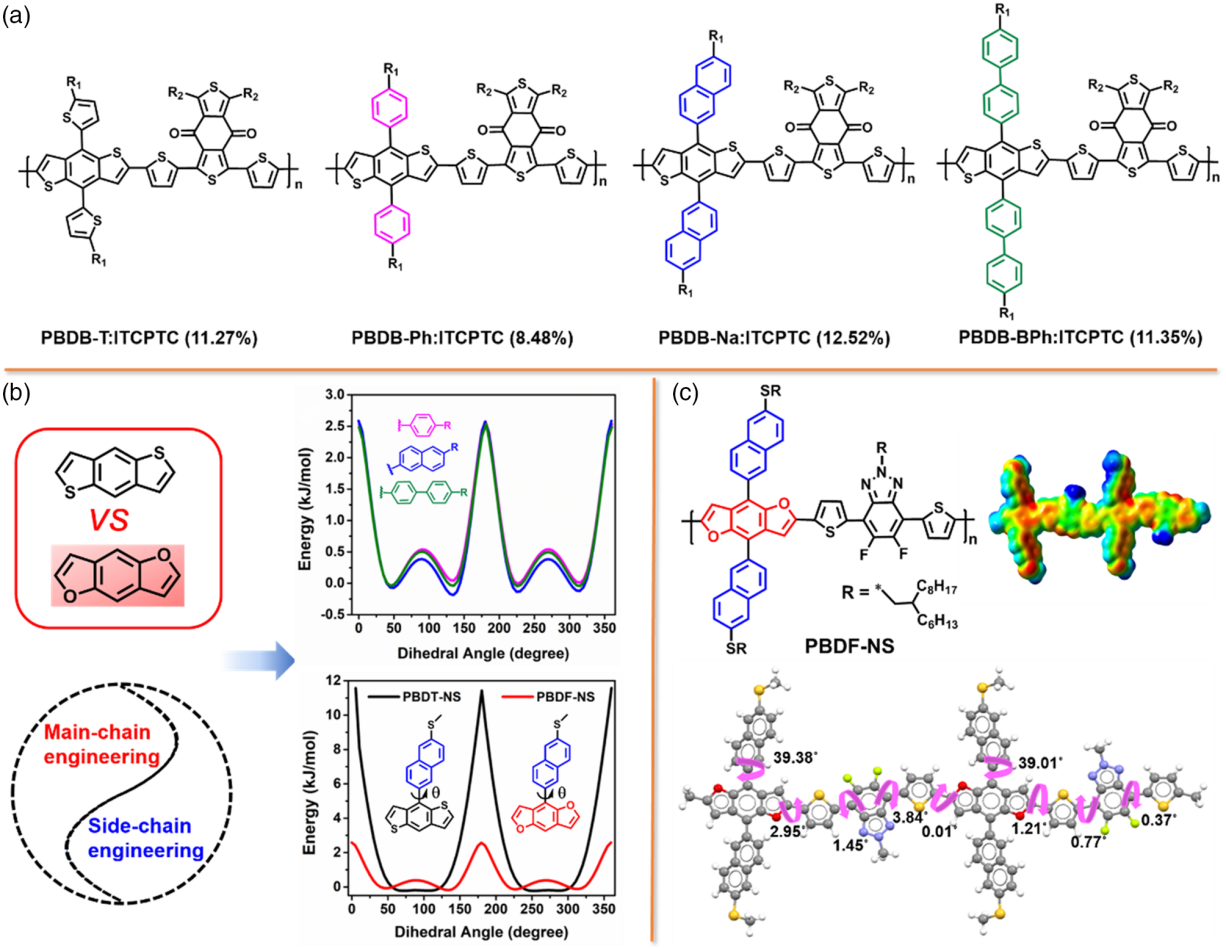
a) Chemical structures of BDT‐based copolymers with different side chains and their photovoltaic performance. b) The twisting barrier of BDT‐based copolymers with different side chains (top) and that of naphthalene‐substituted BDT and BDF units (down). c) Chemical structure of the copolymer PBDF‐NS and its map of the ESP surface and backbone curvature. Reproduced with permission.^[^ ^99^ ^]^ Copyright 2022, Wiley‐VCH.

**Figure 11:**
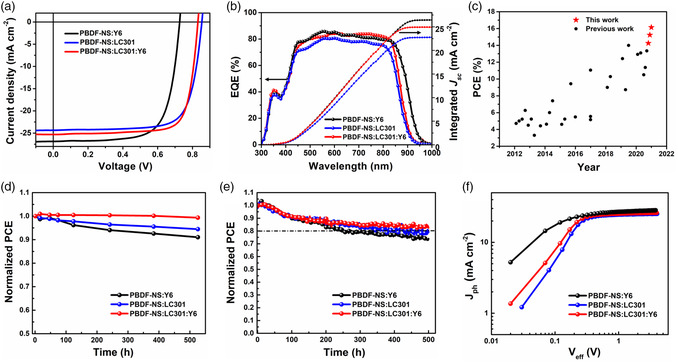
a) Current–voltage (*J*–*V*) characteristics of the binary and ternary devices under simulated AM 1.5G illumination at 100 mW cm^−2^. b) EQE spectra and the integrated *J*_sc_ of the binary and ternary devices. c) PCEs of BDF‐based PSCs based on previous work and this work. d) Normalized PCEs of the storage stability in nitrogen atmosphere. e) Normalized PCEs expressed as a function of light‐soaking time under maximum power point tracking. f) Curves of the photocurrent density (*J*_ph_) versus the effective bias (*V*_eff_) for the optimized devices. Reproduced with permission.^[^ ^99^ ^]^ Copyright 2022, Wiley‐VCH.

Following the merits of less steric hindrance, strong rigidity and fluorescence, good planarity and excellent stacking, high charge carrier mobility, and complementary absorption bands with NFAs in the vis–NIR range, great advances are achieved in BDF polymer‐based PSCs with PCE over 14%. At present, most reported studies of BDF polymer‐based organic solar cells (OSCs) have not adopted the new star acceptor of the Y6 system. The low‐bandgap acceptor Y6 reveals wide‐range absorption property and high absorption coefficient. Moreover, its HOMO and LUMO energy levels are desired to match the energy levels of BDF‐based polymers. In comparison with the traditional A − D − A‐type ITIC and its derivatives, when blending BDF‐based polymers with Y6, the blend films may form well‐organized donor and acceptor domains, which could give rise to small radiative and nonradiative recombination losses as well as low energetic disorder; thus, the application of BDF‐based polymers is very promising in the Y6 system.^[^ ^96^ ^]^ For the abovementioned BDF copolymers, using Y6 to replace m‐ITIC or its derivatives, the fabricated PSCs can provide a reduced energy loss, improved *J*_SC_, and photovoltaic parameters, as shown in **Table** **3**. For example, P‐FP(F13):Y6 device yielded a PCE of up to 13.34% with increased *J*_SC_ and FF compared with that of the device with m‐ITIC acceptor.^[^ ^97^ ^]^ With Y6, the energy loss could also be reduced, and the nonradiative energy loss was as small as 0.23 eV in the P‐FP:Y6 device due to the small energetic disorder of this blend film.^[^ ^98^ ^]^ However, the PCEs with Y6 in BDF polymer‐based PSCs still lag far behind their BDT polymer counterparts. Thus, an intuitive understanding of the relationship between the structural features of BDF polymer and photovoltaic performances is crucially important. Inspired by previous work for BDT‐based copolymers with different side chains (**Figure** **10**a), we designed and synthesized a high‐efficiency polymer donor PBDF‐NS comprising naphthalene‐substituted BDF and fluorinated BTz unit.^[^ ^99^ ^]^ PBDF‐NS possesses a low‐lying HOMO level of −5.44 eV and a wide bandgap of 1.87 eV. From the theoretical calculations with density functional theory (DFT) in Figure 10b, the twisting barrier of the naphthalene‐substituted BDF unit is much smaller compared with that of the BDT unit, which is beneficial to enhancing the crystallization and aggregation in polymer PBDF‐NS. In addition, the PBDF‐NS has a lower molecular electrostatic potential (ESP) distribution value than PBDT‐NS (Figure 10c). Thus, a larger difference in ESP was formed when the polymer PBDF‐NS blended with Y6 and LC301, leading to a stronger intermolecular interaction. As shown in **Figure** **11**, when blending with Y6, PBDF‐NS‐based device exhibited the highest PCE of 14.26%, with a *V*_OC_ of 0.728 V, a large short‐circuit current density (*J*_SC_) of 26.88 mA cm^−2^, and an FF of 72.9%. Due to the upshifted LUMO energy level of LC301 compared with Y6, the PBDF‐NS:LC301 device produced a higher *V*_OC_ of 0.857 V, accompanied by an outstanding PCE of 15.24%. Meanwhile, an enhanced *J*_SC_ of 25.34 mA cm^−2^ and a better FF of 76.1% are achieved by introducing Y6 as a guest acceptor in the LC301‐based device. As a result, a remarkable PCE of 16.14% is obtained, representing the highest efficiency values for BDF polymer‐based OSCs so far (Figure 11c). Furthermore, the binary and ternary devices both display excellent storage and light‐soaking stabilities (Figure 11d,e), attributed to the stable morphology of donor/acceptor blends.

**Table 3:** Optical bandgap, HOMO energy levels, and photovoltaic parameters for PSCs with NFAs Y6 and its derivatives

Polymer	*E*opt g[eV]	HOMO [eV]	*V*_OC_ [V]		*J*_SC_ [mA cm^−2^]	FF [%]	PCE [%]	Ref.
PBF1‐C	1.79	−5.43	0.74	25.21		66.0	12.31	[95]
PBF1‐C‐2Cl	1.97	−5.61	0.83	23.88		66.0	13.10	[95]
F10(P‐FT)	1.93	−5.48	0.75	21.87		65.1	10.61	[93]
L2(F11)	1.86	−5.50	0.77	19.71		70.1	11.03	[93]
P‐P	–	−5.33	0.62	24.20		54.3	9.49	[93]
F13(P–4FP)	1.93	−5.54	0.82	23.27		70.4	13.34	[97]
PBDF‐NS	1.87	−5.44	0.73	26.88		72.9	14.26	[99]
			0.86	23.90		74.4	15.24	[99]
			0.86	25.24		74.6	16.17	[99]

**Figure 12:**
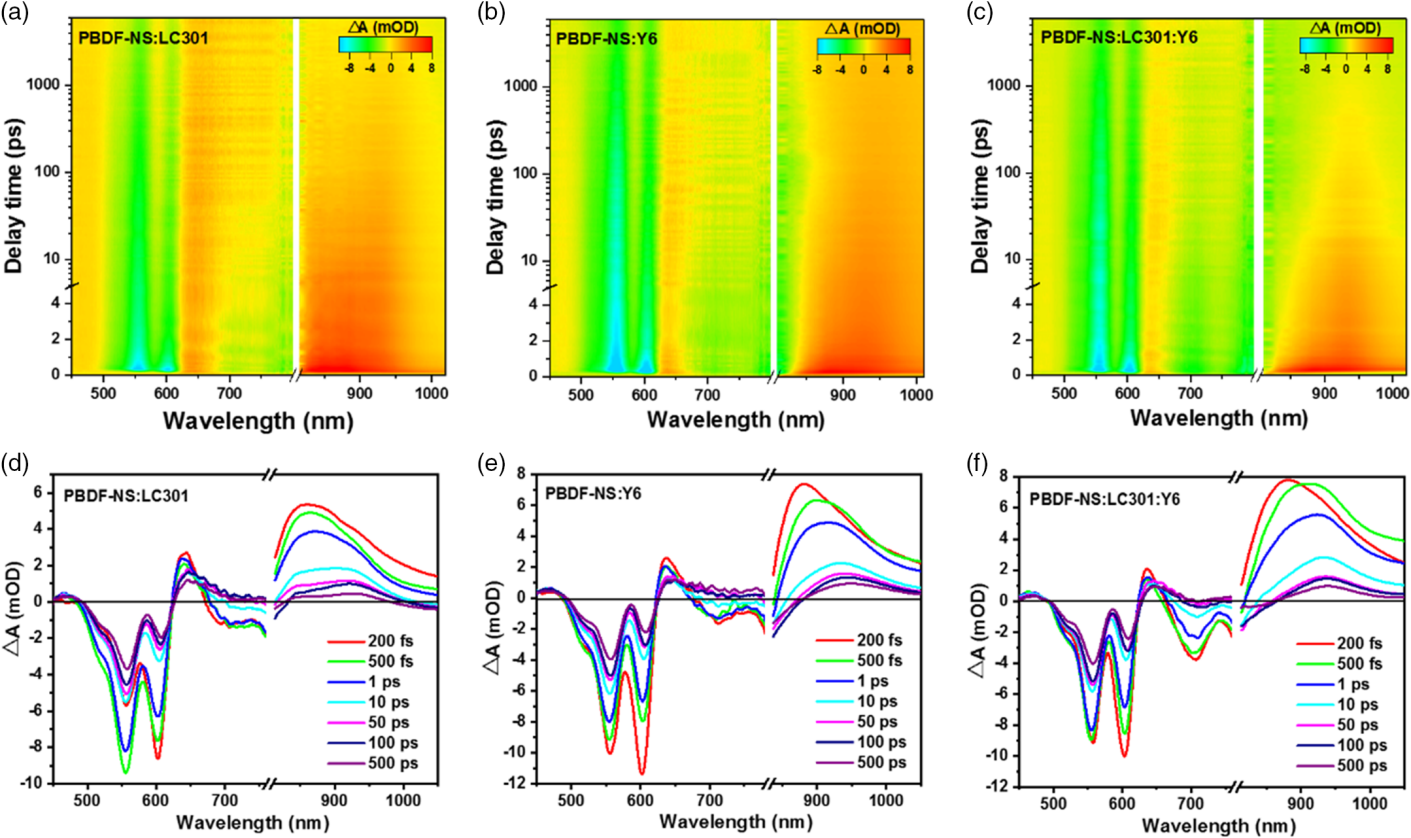
a–c) The 2D TA maps for the binary and ternary blend films pumped at 400 nm. d–f) The spectra recorded at different time delays pumped at 400 nm for the binary and ternary blend films. Reproduced with permission.^[^ ^99^ ^]^ Copyright 2022, Wiley‐VCH.

To explain the significantly improved PCE of PBDF‐NS‐based devices, the photocurrent density (*J*_ph_) versus effective voltage (*V*_eff_) was measured to understand charge generation and dissociation process (Figure 11f). The exciton dissociation probability in ternary PSCs is slightly higher than those of binary devices of Y6 and LC301, suggesting that the ternary blend facilitated charge generation and exciton dissociation. To further investigate the kinetics of charge transfer and charge separation in the active layers, femtosecond time‐resolved transient absorption (fs‐TA) spectroscopy of the blend films was carried out, as shown in **Figure** **12**. Through analyzing the absorption modulation (ΔA) spectra at different delay times, it was found that the electron transfer from donor to acceptor LC301 is inconspicuous in the PBDF‐NS:LC301 blend. However, the fast and efficient electron transfer process occurred in the PBDF‐NS:Y6 blend and ternary blend, which is beneficial for charge generation and achieving a large *J*_SC_. Moreover, PBDF‐NS:PY‐IT device yielded a PCE of 16.17%, which is one of the highest values for all‐PSCs (all‐PSCs) reported to date. It was a first attempt using BDF‐based donor polymers in all‐PSCs. This work provides new thoughts for BDF‐based polymers in all‐PSCs. Taking the advantage of the planar molecular plane of BDF‐based polymers, further rationally regulating crystallinity and molecular packing of polymer donor could obtain ideal nanoscale phase separation in the polymer:acceptor blend. The reported strategies include main‐chain engineering, side‐chain engineering, random copolymerization, etc. It is worth noting that the emergence of high‐performance polymer donors does not happen overnight, it will suffer from long‐term explorations.

## 4 Conclusion and Outlook

In summary, the BDF‐based polymers have made significant progress and obtained eminent photovoltaic performance in NFA‐PSCs. Tailoring the chemical structure of BDF polymer and forming good compatibility between the BDF polymer donor and the acceptor could balance the phase separation and charge transport. Combined with the better understanding for the processing optimization of kinetics and thermodynamics, photophysical phenomena, energy loss, as well as interface engineering, we believe that the PCEs of BDF polymer‐based PSCs will catch up with or even surpass the PCE of BDT‐based polymers in the near future. Thus, to keep pace with BDT polymer‐based PSCs, several challenges must be conquered to further achieve remarkable progress in BDF polymer‐based PSCs. The direction of developing BDF‐based PSCs is proposed.

The molecular structure of the D − A copolymer determines the fundamental properties of the resulting polymer. In the BDF‐containing conjugated polymers, the main‐chain and side‐chain engineering focusing on the atomic substitutes, π‐bridges or various acceptor units will be mainly applied to tune the molecular stacking behavior beside the expected electrochemical and optical properties. Attempts to exploit novel π‐bridges and acceptor units with high absorption coefficients and low molecular ESP are necessary to achieve the high photovoltaic performance of BDF‐based polymers. The molecular aggregation of BDF‐containing conjugated polymers is different with BDT‐containing conjugated polymers at a large content. That said, the general role of molecular optimization strategy in the BDT‐based polymer may not be able to be adopted in BDF‐based polymers directly. The stronger electronegativity and smaller atomic size of oxygen in the furan unit than that of sulfur in the thiophene unit make the BDF‐based polymer chains possess distinct aggregation behavior, which needs more exploration in terms of new BDF polymers to reach a better understanding. In addition, improving the hole mobility of BDF‐containing polymer through the feasible molecular design is also a key point to be considered as it is more related to the FF in PSC, and the current FF values in BDF polymers‐based PSC are also largely behind their BDT counterparts.

The application of BDF‐based polymers in PSCs is monotonous, which has been keeping a blank period in ternary solar cells, tandem solar cells, semitransparent solar cells, and indoor solar cells. The efforts can be devoted in different aspects to explore diverse applications for higher efficiency. Moreover, most BDF‐based polymer donors possess a strong aggregation property, which is adverse to forming a beneficial pure domain and nanoscale phase separation between the polymer donor and the acceptor, leading to poor charge generation and charge transport. Thus, selecting appropriate NFAs, including Y6, Y6 derivatives, and other acceptors, which have suitable bandgaps and energy levels with the BDF polymers, is important to further boost the efficiency of OSCs. A deep understanding of the morphology modulation and the processing condition between the BDF polymer donor and the NFA is necessary. Furthermore, energy loss is one of the main obstacles that limits the PCE improvement. BDF polymer‐based PSCs show relatively larger energy losses compared with those of BDT‐based polymers. Thus, it is important to understand the correlation between *E*_loss_ and the molecular structure of the BDF polymer to further reduce *E*_loss_.

As the current state‐of‐the‐art photovoltaic performance of BDF‐based polymers has been much improved, further explorations of BDF‐containing polymers in other aspects of PSCs can be also considered, such as, 1) applying BDF‐based polymers in all‐PSCs, which have shown great success in our previous work; 2) green solvent‐processed BDF‐based polymers for low‐cost and environment‐friendly PSCs; 3) the quasi layer‐by‐layer PSCs using the BDF‐based polymers; and 4) device stability and their degradation involved with BDF‐based polymers.

## Conflict of Interest

The authors declare no conflict of interest.
